# Wearable and modular functional near-infrared spectroscopy instrument with multidistance measurements at four wavelengths

**DOI:** 10.1117/1.NPh.4.4.041413

**Published:** 2017-08-18

**Authors:** Dominik Wyser, Olivier Lambercy, Felix Scholkmann, Martin Wolf, Roger Gassert

**Affiliations:** a ETH Zurich, Rehabilitation Engineering Laboratory, Department of Health Sciences and Technology, Zurich, Switzerland; b University Hospital of Zurich, Biomedical Optics Research Laboratory, Department of Neonatology, Zurich, Switzerland

**Keywords:** multidistance functional near-infrared spectroscopy, silicon photomultipliers, brain–computer interface, short-channel regression, cytochrome-c-oxidase

## Abstract

With the aim of transitioning functional near-infrared spectroscopy (fNIRS) technology from the laboratory environment to everyday applications, the field has seen a recent push toward the development of wearable/miniaturized, multiwavelength, multidistance, and modular instruments. However, it is challenging to unite all these requirements in a precision instrument with low noise, low drift, and fast sampling characteristics. We present the concept and development of a wearable fNIRS instrument that combines all these key features with the goal of reliably and accurately capturing brain hemodynamics. The proposed instrument consists of a modular network of miniaturized optode modules that include a four-wavelength light source and a highly sensitive silicon photomultiplier detector. Simultaneous measurements with short-separation (7.5 mm; containing predominantly extracerebral signals) and long-separation (20 mm or more; containing both extracerebral and cerebral information) channels are used with short-channel regression filtering methods to increase robustness of fNIRS measurements. Performance of the instrument was characterized with phantom measurements and further validated in human *in vivo* measurements, demonstrating the good raw signal quality (signal-to-noise ratio of 64 dB for short channels; robust measurements up to 50 mm; dynamic optical range larger than 160 dB), the valid estimation of concentration changes (oxy- and deoxyhemoglobin, and cytochrome-c-oxidase) in muscle and brain, and the detection of task-evoked brain activity. The results of our preliminary tests suggest that the presented fNIRS instrument outperforms existing instruments in many aspects and bears high potential for real-time single-trial fNIRS applications as required for wearable brain–computer interfaces.

## 1 Introduction

Functional near-infrared spectroscopy (fNIRS) is an optical technique for the noninvasive measurement of human brain activity.^1^^,^^2^ Near-infrared (NIR) light, emitted from light sources placed on the scalp, travels through the tissues (i.e., scalp, skull, and brain) and is measured as diffusely reflected light by detectors. Based on the attenuation changes of the detected light, relative changes of oxy- ($O_{2}\mathrm{Hb}$) and deoxyhemoglobin (HHb) concentration in the brain region between source and detector can be reconstructed. When a person is mentally active (e.g., during the execution of a motor task), the concentrations of $O_{2}\mathrm{Hb}$ ($\left[O_{2}\mathrm{Hb}\right]$) and HHb ([HHb]) in the brain vary (functional hyperemia due to neurovascular coupling), thereby providing an estimate for local brain activity detectable by fNIRS.

Through the development of fiberless and portable instruments, fNIRS opens new avenues for wearable brain imaging applications.^3^^–^^10^ In particular, wearable fNIRS devices may find application in brain–computer interfaces (BCI) for “out of the lab” applications, e.g., to trigger robotic devices for assistance or rehabilitation of neurological patients in the home environment,^11^^,^^12^ for the communication of locked-in patients,^13^^,^^14^ or for neuroergonomic investigations (i.e., investigating brain behavior in workplace environments).^7^^,^^15^ In comparison with other BCI technologies, fNIRS has the advantage of being small and inexpensive (e.g., compared to magnetic resonance imaging and magnetoencephalography), noninvasive (e.g., compared with electrocorticography), and robust to electrical noise (e.g., compared with electroencephalography). Recently, Ferrari et al.^16^ highlighted that fNIRS hardware development is still in an experimental stage and that there is a need for optimizing and engineering real-world fNIRS instrumentation. By employing commercially available light sources and sensors, fNIRS instruments can be miniaturized relatively easily and manufactured inexpensively.^1^ However, high requirements for signal quality and system reliability make the development of wearable fNIRS instruments challenging.

Several approaches to improve the signal quality of fNIRS instruments have been proposed. Tachtsidis and Scholkmann^17^ recently highlighted the importance of using short-channel regression methods^18^^,^^19^ to remove the influence of systemic physiological changes from the fNIRS signal. Physiological signals are acquired by means of a short-separation (SS) channel [ideally with a source–detector separation (SDS) below 10 mm, as suggested by Refs. 20 and 21], which is then used for filtering the physiological “noise” from long-separation (LS, with SDS typically above 30 mm) measurements, for example, using the filtering approach developed by Saager and Berger.^19^ To the best of our knowledge, no wearable commercial fNIRS instrument currently implements SS channels below 20 mm, precluding short-channel regression approaches for the reduction of physiological noise.

Another approach to maximizing fNIRS signal quality consists of measurements at multiple wavelengths (i.e., more than two) to obtain additional tissue information. Multiwavelength measurements enable the more accurate determination of $\left[O_{2}\mathrm{Hb}\right]$ and [HHb] in the modified Beer–Lambert law (MBLL), since the effect of electronic noise (e.g., switching noise from electronic components, thermal noise, electronic shot noise) is reduced thanks to more observations available for the calculation of the matrix inversion. Furthermore, by including additional wavelengths, the possibility of determining concentration changes of other chromophores is provided. In particular, concentration changes of oxidized cytochrome-c-oxidase (oxCCO)^22^ can be calculated. Since oxCCO is the terminal enzyme in the electron transport chain, it is a direct marker of mitochondrial oxygen consumption, and the amount of oxidation directly reflects the mitochondrial metabolism. Most commercial fNIRS devices employ only two wavelengths,^1^ and only a few prototypes offer the possibility of measuring at additional wavelengths.

To reliably capture signals from the cerebral cortex of the human brain, a large SDS is needed as it allows comparably more light to travel through deeper tissue regions before reaching the detector.^21^ Since at larger distances the light is strongly attenuated (e.g., attenuation of $10^{3}$ to $10^{4}$ at 30 mm SDS^23^), highly sensitive hardware to detect small light intensities is needed.^24^ Many stationary fNIRS devices use avalanche photodiodes or photomultiplier tubes (see Ref. 1 for a review), which exhibit the highest photosensitivity.^25^ However, due to their large size, high cost, and high operating voltages, these technologies are not well suited for wearable instruments. Photodiodes are the most commonly used detector technology for wearable fNIRS instruments^1^ because they operate at low voltages (∼3  V), are inexpensive components, and are small in size. In recent years, the use of silicon photomultipliers (SiPMs) has been exploited for fNIRS applications^5^^,^^24^^,^^26^ due to their high photosensitivity for a small component size and their relatively low cost. In our previous work,^24^ we developed a first prototype of an SiPM-based fNIRS instrument demonstrating a high signal-to-noise ratio (SNR) of more than 70 dB for SDS below 30 mm, as well as a high photosensitivity for small light intensities at larger SDS. While this initial prototype confirmed that SiPMs are highly suitable for wearable fNIRS instruments,^24^^,^^26^^,^^27^ it suffered from different limitations (e.g., size, data readout, lack of modularity, optical drift) that required further improvements and motivated the development of another instrument.

Building on our previous work,^24^ the aim of this project is to design and implement a highly sensitive fNIRS instrument that unites three main features: (i) simultaneous measurements with SS and LS channels, (ii) measurements with four-wavelength light sources, and (iii) modular optode design to accommodate for fNIRS recordings over various brain regions of interest. We expect that an instrument combining these three key features will provide a high-quality fNIRS device that could help bring fNIRS technology “out of the lab” and make it available for a wide range of applications. In the subsequent sections, we present the concept and technical overview of the fNIRS instrument and further report on its preliminary performance evaluation through phantom and *in vivo* measurements.

## 2 Methods

### 2.1 Instrument Requirements

The proposed continuous-wave instrument was developed to fulfill four main aspects, adapted from von Lühmann et al.^6^

i. *Signal quality:* A good raw signal quality, as measured by a high SNR, is crucial for an accurate determination of changes in chromophore concentrations assessed by fNIRS. In general, an SNR above 60 dB (i.e., an average noise magnitude smaller than 1/1000 of the raw signal intensity) should be achieved to allow for appropriate signal quality.^28^ Nevertheless, several studies reported working fNIRS instruments with SNR in the range of 40 dB.^24^^,^^29^^,^^30^ Drift in the raw measurements could lead to undesired concentration divergence of $O_{2}\mathrm{Hb}$ and HHb without physiological origin and should consequently be minimized. A sampling frequency above 6 Hz^3^ is important for accurately resolving the time course of the physiological signals (typically in the range of 0.1 to 1.2 Hz) and decreasing the influence of electronic noise.
ii. *Safety:* The instrument should not pose any risk of injury to the user, neither electrically, thermally, optically, nor mechanically. In particular, the device must contain multiple layers of safety in hardware (current limiters, fuses, emergency switches) and software (overcurrent detection, emergency shutdown) for eliminating the risks of electric failure. The maximal temperature of components in contact with the skin should not exceed 42°C to prevent thermal injuries to the skin.^31^ The maximal optical power for the uncollimated and incoherent light of light-emitting diodes (LEDs), which will be used in the proposed fNIRS instrument, is typically below 10 mW.^3^^,^^4^^,^^24^^,^^32^ Finally, any harm from mechanical pressure (e.g., due to optode placement) must be prevented.
iii. *Usability:* For minimizing motion artefacts and discomfort during measurements, the weight of the device must be small.^33^ To achieve higher acceptance by the user, a comfortable optode placement must be addressed by guaranteeing an unobtrusive and robust optode attachment on the scalp, in addition to having a user-friendly graphical user interface (GUI) for simple and fast operation of the instrument.
iv. *Modularity:* To address versatile user needs regarding different experimental protocols/questions, the number of channels must be scalable and individually selectable. To cover a brain area of interest (e.g., the primary motor cortex) and surrounding tissues, measurements with an eight sources and eight detectors setup^3^ should be achieved. To ensure a high SNR at all SDS, the emitted light intensities and the detector sensitivity must be adjustable by software.

### 2.2 System Description

**Fig. 1:**
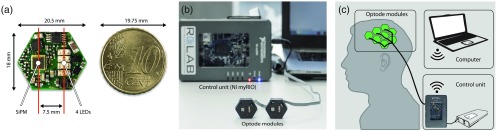
Visualization of the developed fNIRS instrument. (a) Close-up of the PCB next to a 10-cent euro coin. (b) Picture of the fNIRS instrument with two custom-made optode modules in rapid prototyped casings (front), the control unit, and a PC. (c) Conceptual sketch illustrating the arrangement of the system with eight modules placed over the motor cortex of an adult human. Optode modules are connected to a control unit (NI myRIO and a battery) allowing for wireless communication with a laptop computer.

The concept of the proposed fNIRS instrument (Fig. 1) consists of a reconfigurable network of small hexagonal modules with on-board electronics, as well as emitters (four LEDs) and a detector (SiPM). Each individual module can be operated separately and can be combined with other modules, allowing the realization of both SS (7.5 mm) and various LS (20 mm or more) measurement channels. Optode modules are controlled by a control unit that can be placed remotely and that is connected to a personal computer (PC). A freely selectable number of optode modules can be used simultaneously and in a way to optimally cover the area of interest on the subject’s head.

A GUI programmed in LabVIEW [National Instruments (NI), Texas] operates the measurement from any computer containing a wireless receiver. The control unit consists of a power supply and a NI myRIO embedded controller. The NI myRIO performs all data processing steps and simultaneously acts as the interface between the computer and the optode modules, including Wi-Fi communication with the computer and $I^{2}C$ (inter-integrated circuit) data transfer with the modules. A battery provides electrical power for the NI myRIO (12 V) and the optode modules (3.6 V).

The optode modules and the control unit are connected by one cable with five lines: power (3.6 V, GND), communication ($2\times I^{2}C$), and timing (SYNC; used for timed measurements). Each optode module contains four LEDs and one SiPM. Alternatively, one module of the connected modules operates as source and all the others as detector—including the source module itself— enabling simultaneous measurements with SS [7.5 mm, representing the physical distance between LEDs and SiPM on the printed circuit board (PCB)] and LS channels. Modularity is achieved by being able to add and remove modules between measurement sessions.

### 2.3 Hardware Design of SiPM-Based Optode Modules

**Fig. 2:**
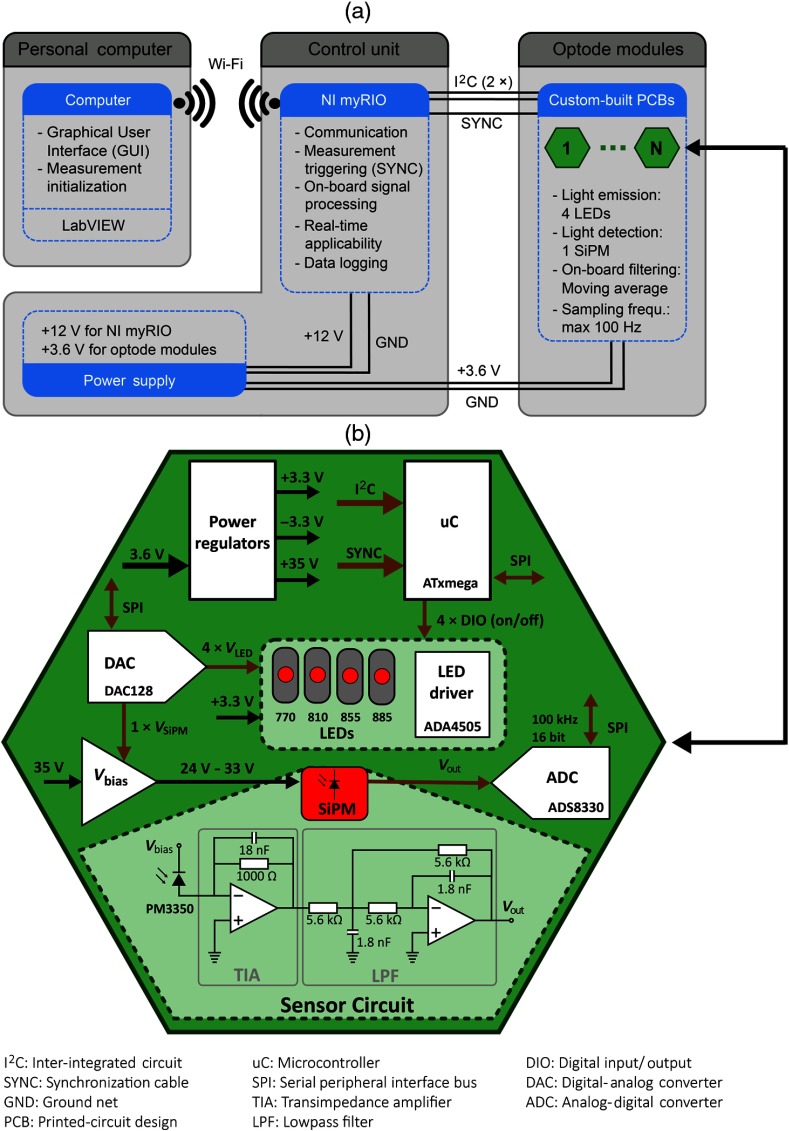
Schematic diagram of the fNIRS instrument. (a) The three main components of the instrument, including a computer, a control unit consisting of the NI myRIO, and a battery, as well as N optode modules. (b) Diagram showing the electrical components of the custom-built optode modules. Black arrows represent power lines, and brown arrows represent signal lines.

**Fig. 3:**
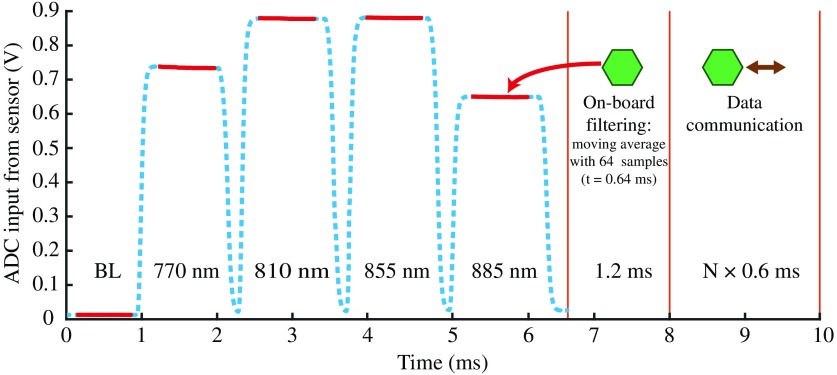
Visualization of the timing of one measurement cycle. One module is set as source, time-multiplexing four wavelengths. At the level of each detector, the measured signal (blue line, dashed) is composed of a series of voltage step impulses corresponding to the signals obtained from each LED of the source module. For each step impulse, on-board data averaging of 64 acquired samples corresponding to the plateau (red line, 0.3 ms after powering the LED) is performed. The data acquisition phase (including a baseline backlight measurement BL) lasts 6.6 ms, whereas data processing requires another 1.2 ms. Data communication ($I^{2}C$) between one optode module and the NI myRIO lasts 0.6 ms.

The PCBs of the optode modules contain all electrical components for individual fNIRS data acquisition (light emission and detection; see Fig. 2, bottom), including power regulators, microcontroller (μC), digital-analog convertor (DAC), and analog-digital convertor (ADC). The PCBs were manufactured based on the rigid-flex PCB technology, where specific parts of the PCBs have flexible properties that can be bent, allowing the omission of delicate board-to-board connectors. One PCB consists of two rigid hexagons that are connected by one flexible connecting part.

i. *Power*: Each optode module is supplied with a voltage of 3.6 V, corresponding to a lithium-ion polymer battery. A fuse on the power net (3.6 V) prevents currents above 300 mA. The supply voltage is converted into three voltages of +3.3, +35, and −3.3  V, and, for every power net, linear dropout (LDO) regulators are applied. The +3.3  V is the main operating voltage of the modules, separately powering the decoupled digital (i.e., microcontroller) and analog components (i.e., LEDs, ADC, DAC, operational amplifiers). The +35  V is used to generate the overvoltage of the SiPM. To prevent excess current flow through the SiPM and overheating, a current limiter before the LDO regulator becomes active if the current flow through the sensor exceeds 4 mA. The −3.3  V is used for the negative supply voltage of the operational amplifiers.
ii. *LED*: Each module is equipped with four LEDs (OIS-330, OSA Opto Light GmbH, Berlin, Germany) at wavelengths of 770±8  nm, 810±10  nm, 855±15  nm, and 885±15  nm (see Table 1). The wavelengths were selected according to Arifler et al.^34^ who investigated the ideal wavelength-combination to calculate $\left[O_{2}\mathrm{Hb}\right]$, [HHb], and [oxCCO] to minimize chromophore crosstalk and maximize separability.^35^^,^^36^ The LEDs are manufactured with a lens on top of the chip for small view angles around 40 deg. The actual wavelengths measured with a spectrometer (Maya2000, Ocean Optics, Ostfildern, Germany) at a forward current of $I_{F}=30\;\mathrm{mA}$ were found to be 774, 817, 865, and 892 nm. The full-width at half-maximum (FWHM) values ranged between 35 and 51 nm and the emitted power at $I_{F}=30\;\mathrm{mA}$ [measured with a power meter (ILX Lightwave OMM-6810B, Newport, California)] between 2 and 7.2 mW.
  The LEDs are operated in a time-multiplexed manner (Fig. 3) and are powered in sequence for 1.2 ms each. For each LED separately, the output of an operational amplifier (ADA4505, Analog Devices Inc., Massachusetts) adjusts the base of a transistor regulating the voltage drop over a shunt resistor (for more details see Circuit Note CN0125 by Analog Devices, Massachusetts). Depending on the DAC output ($4\times V_{\mathrm{LED}}$) that is fed to the amplifier’s positive input, the LED’s forward current $I_{F}$ can be tuned between 1 and 90 mA. All LEDs can be separately turned on/off with four DIOs (digital input/output) of the microcontroller, allowing for fast switching. For all measurements presented in this publication, the forward currents were adjusted for leveled detector intensities, with values of 30 (770 nm), 60 (810 nm), 30 (855 nm), and 90 mA (885 nm).
iii. *Sensor circuit*: Every module contains one SiPM for light detection (PM3350, Ketek GmbH, Munich, Germany). In the proposed setup, the sensitivity of the SiPM is adjusted by changing its overvoltage at the beginning of the measurement; a low overvoltage allows the measurement of a large number of photons at short SDS, whereas a high overvoltage allows the detection of a very small number of photons at large SDS. Internal heating of the SiPM was minimized by limiting the photocurrent to 1.5 mA in software.
  The SiPM has an active area of $3\times 3\;{\mathrm{mm}}^{2}$, contains 3600 cells (50  μm size) operating in Geiger mode, and has an internal gain of $8.2\times 10^{6}$. SiPMs have shown to be linear for longer SDS above ∼30  mm,^26^ but for shorter distances in the range where the detector saturates, compensation strategies (i.e., calibration curves from *a priori* measurements) can be applied to account for the nonlinear behavior. The operating voltage of the SiPM is generated by down-regulating +35  V to a voltage in the range between +24 and +33  V ($V_{\text{bias}}$). To do so, the feedback loop of an LDO regulator (TPS7A4901, Texas Instruments, Texas) is controlled by an analog output from the DAC ($V_{\mathrm{SiPM}}$). At +24  V, the sensor is operated below breakdown voltage (no light detection, sensor gain G=0), becoming gradually more sensitive by increasing the voltage to 28 V for the shortest channels ($G=1.4\times 10^{6}$) and to +33  V for the longest channels ($G=8.2\times 10^{6}$). The photon detection efficiencies (PDE) for the four wavelengths are 13%, 9%, 6%, and 4%. The adjustment from maximal (+33  V) to minimal (+24  V) intensity, or vice versa, between two samples/channels takes 3 ms to stabilize.
  The photocurrent through the SiPM is transformed into a voltage between 0 and 3 V with a transimpedance amplifier (TIA). The amplification of the TIA is fixed at −1000, and a capacitor of 18 nF is added to increase stability of the TIA. An analog lowpass filter (LPF) after the TIA with a cutoff frequency of 15.8 kHz removes high-frequency electronic noise. The LPF filter is arranged in a multiple-feedback architecture to act as an antialiasing filter, fulfilling at the same time the function of signal inversion to generate a positive output signal ($V_{\text{out}}$).
iv. *DAC*: The outputs of the 12-bit DAC (DAC128S085, Texas Instruments) are used to tune the feedback loop of the SiPM for sensitivity adjustments and to set the forward currents of the four LEDs. For every channel, the operating voltage of the SiPM is adjusted separately.
v. *ADC*: The ADC (ADS8330, Texas Instruments) has a resolution of 16 bits for measuring the output voltage of the SiPM. The ADC sampling rate is set to 100 kHz, acquiring 660 samples during the 6.6-ms measurement time (Fig. 3). Serial peripheral interface (SPI) communication between ADC and microcontroller allows for fast data readout.
vi. *Microcontroller:* Every module contains its own microcontroller (ATxmega128A4U, Atmel, California) that enables individual operation and simple on-board data processing. After data acquisition (Fig. 3, blue line), the influence of electronic noise is reduced by applying a moving average filter with a window size of 64 samples (red line). The time to perform the averaging of the five subsamples (1 × backlight, 4 × wavelengths) on the microcontroller lasts 1.2 ms.

**Table 1:** Measured LED properties at $I_{F}$ = 30 mA.

Selected wavelength (nm)	Measured wavelength (nm)	FWHM (nm)	Power (mW)
770	774	37	3.5
810	817	35	2.6
855	865	42	7.2
885	892	51	2

Note: $I_{F}$, LED forward current; FWHM, full-width at half-maximum.

The microcontroller communicates with the ADC and DAC via SPI and with the control unit via $I^{2}C$. For $I^{2}C$ communication, each module is given an individual address and communication is performed sequentially for every module. To keep communication time low, only 10 bytes are transmitted per sample and module—two for every wavelength and backlight—resulting in a communication time of ∼0.6  ms per module (Fig. 3). In total, one entire measurement cycle lasts less than 10 ms, including light measurement (6.6 ms), on-board data processing (1.2 ms), and data communication with the control unit (N×0.6  ms), where N is the number of modules that have to be read-out. A maximal overall sampling frequency of 100 Hz can be achieved.

In addition to the hardware safety mechanisms, software safety features are implemented at the level of the microcontroller. If the maximal current through the SiPM exceeds a predefined limit (i.e., 1.5 mA), the sensor is turned off by reducing the operating voltage below breakdown voltage, resulting in the deactivation of the channel. At the same time, all other channels can continue operating as before.

### 2.4 Calculation of Concentration Changes

Concentration changes of oxyhemoglobin, deoxyhemoglobin, and oxCCO (i.e., $\left[O_{2}\mathrm{Hb}\right]$, [HHb], and [oxCCO], respectively) were obtained by applying MBLL for four wavelengths with 774, 817, 865, and 892 nm (UCL4 algorithm^37^). The algorithm was implemented for the reconstruction of two ($\left[O_{2}\mathrm{Hb}\right]$, [HHb]), and three chromophores ($\left[O_{2}\mathrm{Hb}\right]$, [HHb], and [oxCCO])

$$[\begin{array}{c} \Delta [O_{2}\mathrm{Hb}]\\ \Delta [\mathrm{HHb}] \end{array}]=\frac{1}{L}{\left[\begin{array}{cc} \epsilon_{O_{2}\mathrm{Hb}}(\lambda_{1}) & \epsilon_{\mathrm{HHb}}(\lambda_{1})\\ \epsilon_{O_{2}\mathrm{Hb}}(\lambda_{2}) & \epsilon_{\mathrm{HHb}}(\lambda_{2})\\ \begin{array}{c} \epsilon_{O_{2}\mathrm{Hb}}(\lambda_{3})\\ \epsilon_{O_{2}\mathrm{Hb}}(\lambda_{4}) \end{array} & \begin{array}{c} \epsilon_{\mathrm{HHb}}(\lambda_{3})\\ \epsilon_{\mathrm{HHb}}(\lambda_{4}) \end{array} \end{array}\right]}^{-1}\times [\begin{array}{c} \Delta A(\lambda_{1})/\mathrm{DPF}(\lambda_{1})\\ \Delta A(\lambda_{2})/\mathrm{DPF}(\lambda_{2})\\ \begin{array}{c} \Delta A(\lambda_{3})/\mathrm{DPF}(\lambda_{3})\\ \Delta A(\lambda_{4})/\mathrm{DPF}(\lambda_{4}) \end{array} \end{array}],$$ (1)

$$[\begin{array}{c} \Delta [O_{2}\mathrm{Hb}]\\ \Delta [\mathrm{HHb}]\\ \Delta [\mathrm{oxCCO}] \end{array}]=\frac{1}{L}{\left[\begin{array}{ccc} \epsilon_{O_{2}\mathrm{Hb}}(\lambda_{1}) & \epsilon_{\mathrm{HHb}}(\lambda_{1}) & \epsilon_{\mathrm{oxCCO}}(\lambda_{1})\\ \epsilon_{O_{2}\mathrm{Hb}}(\lambda_{2}) & \epsilon_{\mathrm{HHb}}(\lambda_{2}) & \epsilon_{\mathrm{oxCCO}}(\lambda_{2})\\ \epsilon_{O_{2}\mathrm{Hb}}(\lambda_{3}) & \epsilon_{\mathrm{HHb}}(\lambda_{3}) & \epsilon_{\mathrm{oxCCO}}(\lambda_{3})\\ \epsilon_{O_{2}\mathrm{Hb}}(\lambda_{4}) & \epsilon_{\mathrm{HHb}}(\lambda_{4}) & \epsilon_{\mathrm{oxCCO}}(\lambda_{4}) \end{array}\right]}^{-1}\times [\begin{array}{c} \Delta A(\lambda_{1})/\mathrm{DPF}(\lambda_{1})\\ \Delta A(\lambda_{2})/\mathrm{DPF}(\lambda_{2})\\ \begin{array}{c} \Delta A(\lambda_{3})/\mathrm{DPF}(\lambda_{3})\\ \Delta A(\lambda_{4})/\mathrm{DPF}(\lambda_{4}) \end{array} \end{array}].$$ (2)

Extinction coefficients $\epsilon_{C}(\lambda_{i})$ for the chromophore C and wavelength $\lambda_{i}$ were selected according to Matcher et al.^37^ Normalized values for the differential pathlength factor (DPF) were obtained from the Biomedical Optics Research Group at University College London, renormalized according to Ref. 38, and multiplied with the physical channel length L. Since the concentration of oxCCO in the cells is assumed to be constant, the difference spectrum between oxidized and reduced cytochrome-c-oxidize was used (i.e., oxCCO and redCCO). The inverse of the nonsymmetric extinction matrix E was calculated based on the Moore–Penrose pseudoinverse $E=E^{\prime }{\left(EE^{\prime }\right)}^{-1}$. The change of optical density is defined as $\Delta A=-\ln[\frac{I(\lambda_{i},t)}{I(\lambda ,t_{0})}]$, where I(λ,t) and $I(\lambda ,t_{0})$ are the optical signal measured by the sensor for the current and the previous sample, respectively.

Short-channel regression was implemented according to Saager and Berger.^19^ It is assumed that the SS channel contains only signal contributions of extracerebral physiological origin and that it can be subtracted with a scaling factor from the LS measurement, which contains a combination of extracerebral and cerebral signals. The scaling factors for $O_{2}\mathrm{Hb}$ and HHb are both obtained from the correlation between the long and short channels.

### 2.5 Experimental Protocol for Instrument Evaluation

The proposed fNIRS instrument was tested by means of phantom and *in vivo* measurements to evaluate the ability to reliably measure hemodynamic changes. First, measurements on silicone phantoms mimicking human tissue were performed to evaluate the technical performance of the instrument. Subsequently, *in vivo* measurements on muscle and brain tissue were performed to characterize the instrument’s performance in reconstructing $\left[O_{2}\mathrm{Hb}\right]$, [HHb], and [oxCCO].

#### 2.5.1 Phantom measurements

A silicone phantom (ISS Inc., Champaign, Illinois) with similar optical properties as the tissues of the human forehead was selected for the measurements (Table 2).^11^ The optical properties of the silicone phantom were obtained with a frequency-domain fNIRS system (ISS OxiplexTS). The optical loss (OL) in the phantom denotes the inverse value of the reflectance (units: ${\mathrm{mm}}^{-2}$) multiplied with a standardized circular aperture of 8 mm according to the IEC 80601-2-71:2015 standard. Reflectance was obtained by solving the diffusion equation for a semi-infinite medium,^39^ which was validated with Monte-Carlo simulations,^40^ as well as reference measurements performed with a power meter (ILX Lightwave OMM-6810B). In the phantom, the scattering behavior (quantified by the reduced scattering coefficient $\mu_{s}^{\prime }$) is slightly stronger than that of the human forehead tissue, whereas the absorption coefficient ($\mu_{a}$) is similar; this results in a slightly stronger overall light attenuation of the silicone phantom compared with the human head tissue. Thinking of future fNIRS applications investigating motor-related brain areas under hairy regions, the overall light attenuation of the phantom is in a realistic range.

**Table 2:** Optical properties of a human forehead and the optical silicone phantom used for the validation measurements.^24^

Tissue	$\mu_{a,680}$ (${\mathrm{mm}}^{-1}$)	$\mu_{a,850}$ (${\mathrm{mm}}^{-1}$)	$\mu_{s,680}^{\prime }$ (${\mathrm{mm}}^{-1}$)	$\mu_{s,850}^{\prime }$ (${\mathrm{mm}}^{-1}$)
Human forehead	0.0109	0.0115	0.8235	0.7210
Silicone phantom	0.0104	0.0099	1.1093	0.9514

High-quality raw signals are crucial in fNIRS for optimal spectroscopic separation of the chromophores and to achieve a high repeatability and reproducibility of the measurement. For validation of signal quality, different performance features were evaluated to obtain an overview of the instrument’s performance:

i. *SNR:* Noise in the raw signal affecting chromophore calculation was evaluated by calculating the SNR of the raw light intensities. SNR was calculated as $20\;\log_{10}(\overset{¯}{s}/s_{\sigma })$, with mean value $\overset{¯}{s}$ and standard deviation $s_{\sigma }$. Measurements on the silicone phantom were obtained for SDS between 7.5 and 65 mm at a sampling frequency of 100 Hz with the identical protocol as in Ref. 24. In the phantom used for these measurements, OL of $10^{4}$, $10^{6}$, and $10^{7}$ corresponds to ∼25, 45, and 55 mm, respectively.
ii. *Temperature drift:* To reduce heating effects in the SiPM leading to drift, two possibilities exist: either the duty cycle of the measurement or the photocurrent flowing through the sensor can be reduced. To maintain a high temporal resolution, we limited the photocurrent to 1 mA. Drift was measured by calculating the slope (linear fit) for a 1-min window, 10 min after measurement start. Temperature information was simultaneously acquired with a heat-camera FLIR E5 (FLIR Systems, Oregon). For faster warming up, a warm up routine that performs a continuous LED emission at forward current of $I_{F}=5\;\mathrm{mA}$ with simultaneous SiPM measurement for 60 s was implemented.
iii. *Optical sensitivity:* It is a measure of the minimal light intensity that an optical instrument can detect. It is defined as the noise equivalent power (NEP) where photocurrent and noise are of equal magnitude (SNR=0  dB). The NEP was found by measuring the dark noise for 1000 samples in total darkness with the light sources turned off. The obtained photocurrent was transformed into an optical power by means of the SiPM characteristics (gain $G=8.2\times 10^{6}$ and PDE=13%, 9%, 6%, and 4%).
iv. *Dynamic optical range:* It is defined as the ratio of the maximal measurable power and the minimal detectable optical power. Since the operating voltage of the SiPM can be adjusted to the incident light intensities, saturation effects can be prevented, enabling measurements of relatively high optical power. Accordingly, the maximal measurable power was set to 1 mW for all wavelengths. The NEP values were used from the previous measurement on optical sensitivity. The dynamic range is given in decibel, where 20 dB corresponds to one decade.
v. *Variance of reconstructed signal:* To investigate the benefit of including additional wavelengths (three or four instead of two wavelengths) for chromophore concentration calculation, the variation of the reconstructed concentration changes was investigated. $\left[O_{2}\mathrm{Hb}\right]$ and [HHb] were calculated for eleven detrended steady-state phantom measurements of 400 samples using Eq. (1). The ratio of variance between the two- (770 and 850 nm) and the three- (770, 810, and 850 nm) or four-wavelength (770, 810, 850, and 885 nm) reconstructions was calculated as $\frac{\mathrm{var}({\left[C\right]}^{3wl})}{\mathrm{var}({\left[C\right]}^{2wl})}$ and $\frac{\mathrm{var}({\left[C\right]}^{4wl})}{\mathrm{var}({\left[C\right]}^{2wl})}$, respectively, where [C] represents $\left[O_{2}\mathrm{Hb}\right]$ or [HHb]. The variance of the reconstructed signal is expected to decrease using additional wavelengths, since more tissue information to solve the least squares inverse problem is available.

#### 2.5.2 Physiological measurements

**Fig. 4:**
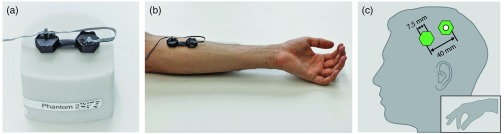
Experimental setup to validate the fNIRS instrument; two optode modules were connected together with a rapid-prototyped element that ensures an SDS distance of 40 mm. (a) Validation measurements performed on a silicone phantom. (b) Occlusion measurement on the left forearm. (c) Placement of two modules next to C3 (white circle) for measuring cerebral hemodynamics from primary motor cortex during a finger-pinching task.

Further evaluation of the instrument was performed during measurements on two human subjects to confirm the ability of the sensor to measure hemodynamic changes with correct trend and magnitude. All subjects were informed about the procedure and gave consent prior to the measurements.

i. *Arterial occlusion:* To evaluate the ability of the system to determine changes in $\left[O_{2}\mathrm{Hb}\right]$, [HHb], and [oxCCO] based on the four selected wavelengths, an arm arterial occlusion measurement was performed.^37^^,^^41^ During the occlusion, the trends of $\left[O_{2}\mathrm{Hb}\right]$ and [HHb] are expected to simultaneously decrease and increase, respectively, while, after pressure release, a strong hyperemic change in the opposite direction should occur.^42^ It is expected that changes in [oxCCO] should remain small, ideally showing a small negative decay.^37^ The measurement protocol was adopted from Ref. 24 with 120 s pressure application (using a manual cuff, 240 mmHg pressure) followed by 180 s rest, with three repetitions (total time: 1020 s). Two optode modules were placed along the flexor digitorum profundus muscle on the left forearm,^41^ at a fixed SDS of 40 mm. A constant SDS was ensured using a rapid-prototyped black casing that connects the two modules with a bendable bridge (Fig. 4). The optode modules were held in place with a Velcro strap and obscured with a black cloth to prevent stray light from affecting the measurement. The overall sampling rate was 50 Hz with two modules (corresponding to 25 Hz per channel). A digital zero-phase LPF (Chebyshev II third order) with a cutoff frequency of 1 Hz was applied to the concentration changes.
ii. *Task-evoked brain activity (motor execution task):* The ability to detect changes in cerebral hemodynamics and oxygenation associated with brain activity can be shown by measuring concentration changes in the primary motor cortex during a motor execution task.^43^^,^^44^ A pilot test with one subject (male, age 51 years) was performed with a left-hand pinching task where the subject was asked to alternatively touch the first, third, second, and fourth finger with his thumb at a self-paced speed.^45^ Two modules were placed over the contralateral primary motor cortex at an SDS of 40 mm [Fig. 4(c)], with the center of the long optical channel (LS) 10 mm frontal and 20 mm inferior of the C3-point from the 10–20 system (according to Toronov et al.,^46^ channel 5 and 6). The protocol was adapted from Toronov et al.^46^ with a task and rest duration of 20 s each and was repeated 10 times. The modules were fixed with Velcro tape and ambient light in the room was dimmed. To prevent artifacts from muscle movements, the subject was asked to not move the head and not speak during the entire measurement. Data were sampled at 50 Hz with two modules, corresponding to 25 Hz per channel, and the SS and LS combinations presenting the strongest signal change were used for the analysis. Noise was removed by means of a fourth-order bandpass Chebyshev II filter (zero-phase) between 0.01 and 0.5 Hz. Changes in $\left[O_{2}\mathrm{Hb}\right]$, [HHb], and [oxCCO] were group averaged for the 10 trials for the long and short channels. Short-channel regression was performed according to Saager and Berger^19^ with a sliding window of 120 s.

## 3 Results

### 3.1 Hardware Characterization and Phantom Measurements

An overview of the technical performance of the fNIRS instrument with focus on the custom-built optode modules is provided in Table 3. A tradeoff between signal quality, sampling frequency, heating, safety restrictions, and component size was found. The modules were manufactured from rigid-flex PCBs, which allowed the miniaturization of the modules to a size of $20.5\times 18\times 8\;{\mathrm{mm}}^{3}$ (PCB only without mechanical casing) or $25\times 22\times 10\;{\mathrm{mm}}^{3}$ (including mechanical casing), and a corresponding weight of 3 and 5 g, respectively. The four wavelengths at 770, 810, 850, and 885 nm were selected to closely match the optimal wavelength obtained from Arifler et al.^34^ to minimize optical crosstalk when calculating $\left[O_{2}\mathrm{Hb}\right]$, [HHb], and [oxCCO]. The power consumption of the modules depends on the emitted light intensity, the photocurrent, and the sampling frequency; in the condition where the maximal power consumption is expected (when measuring at 100 Hz), 0.4 W was not exceeded. By limiting the photocurrent to 1 mA, the temperature of the sensor—being the hottest part of the setup—remained below 41°C.

**Table 3:** Technical performance of the fNIRS instrument.

**Physical properties**		
Light source	4 LEDs (770, 810, 850, 885 nm)	
Light detector	1 SiPM	
SDS	SS: 7.5 mm	
	LS: >20 mm	
Size of optode modules	PCB only: $20.5\times 18\times 8\;{\mathrm{mm}}^{3}$	
	PCB and casing: $25\times 22\times 10\;{\mathrm{mm}}^{3}$	
Weight of optode modules	PCB only: 3 g	
	PCB and casing: 5 g	
**Performance**		
Max power consumption per module (at 100 Hz)	<0.4 W	
Max temperature	≤41°C	
Channel number for n modules	N×N	
Max sampling frequency for $N_{S}$ sources and $N_{D}$ detectors	$100\;\mathrm{Hz}/[N_{S}\times (1+0.06\times N_{D})]$	
**Sensor**		
SNR for OLs (In the phantom used for these measurements, the OLs correspond to SDS of 25, 45, and 55 mm)	$<10^{4}$ OL	64 dB
	$10^{6}$ OL	47 dB
	$10^{7}$ OL	31 dB
NEP (770, 810, 850, 885 nm)	0.94, 1.3, 1.86, 2.67 pW	
Dynamic optical range	>160 dB	
Optical drift of raw intensity signal	After 60 s: ≤0.3‰/s	
	After 600 s: ≤0.1‰/s	

The optical sensitivity of the sensor circuitry, expressed by the NEP, was found to be 0.94, 1.3, 1.86, and 2.67 pW for the four wavelengths. By calculating the ratio of the maximal measurable optical power (1 mW) and the NEP, dynamic ranges larger than 160 dB for all wavelengths are obtained, enabling simultaneous measurements in the order of milliwatt to picowatt. For OL larger than $10^{4}$, the SNR results were identical to our previous work,^24^ with values gradually dropping from 64 to 20 dB for SDS between 35 and 65 mm on the used silicone phantom. The threshold at which the SNR falls below 40 dB is at ∼50  mm (OL $5\times 10^{6}$) for the investigated phantom, LED power, and sampling frequency. For shorter distances, the SNR is in the range of 64 dB.

While at beginning of operation (after 60 s) a visible drift was observed with a negative slope of 0.3 ‰/s or smaller (dependent on the SDS), this value continuously decreased to 0.1 ‰/s after 10 min. For smaller overvoltages (short distances), temperature changes have a stronger influence on the PDE, leading to slightly higher drift coefficients.

Measurements with one or multiple channels are enabled. The total number of channels scales with the number of modules N—there is always a maximum of N×N channels (e.g., 2×2=4 channels when using 2 modules). The sampling frequency is shared across the $N_{S}$ source modules and $N_{D}$ detector modules by $100\;\mathrm{Hz}/[N_{S}\times (1+0.06\times N_{D})]$, where 0.06 is 1/10 of the 0.6 ms communication time that is required for data readout (Fig. 3). For example, for a configuration with 10 modules, where every module alternately operates as a source module, 100 optical channels sampled at 6.25 Hz can be achieved. When two instead of four wavelengths are used, the sampling frequency could be further increased to 125 Hz—the frequency does not double as some steps (e.g., backlight measurement, voltage adjustment) are performed for every sample, independent of the number of wavelengths used. The influence of electronic crosstalk between modules is negligible thanks to specific on-board electronics for the LEDs and SiPM, which are placed on each optode module.

Table 4 highlights the changes in chromophore concentration variance when more than two wavelengths are used for calculating $\left[O_{2}\mathrm{Hb}\right]$ and [HHb]. While the variance is reduced by 6% for [$O_{2}\mathrm{Hb}$] and 1% for [HHb] when three wavelengths are used, the effect gets larger by including a fourth wavelength. $\left[O_{2}\mathrm{Hb}\right]$ shows a reduction of variance by 25% when using four instead of two wavelengths, while the variance of [HHb] decreases by a factor of 8%.

**Table 4:** Decrease of signal variance when additional wavelengths are used for the calculation of $\left[O_{2}\mathrm{Hb}\right]$ and [HHb]. For both chromophores, two wavelength-combinations were investigated (left row), showing the variance decrease in the right row.

Wavelength-combination	Δ variance (%)
$\left[O_{2}\mathrm{Hb}\right]$: two- versus three-wavelengths	−6
$\left[O_{2}\mathrm{Hb}\right]$: two- versus four-wavelengths	−25
[HHb]: two- versus three-wavelengths	−1
[HHb]: two- versus four-wavelengths	−8

### 3.2 Physiological Validation

#### 3.2.1 Arterial occlusion

**Fig. 5:**
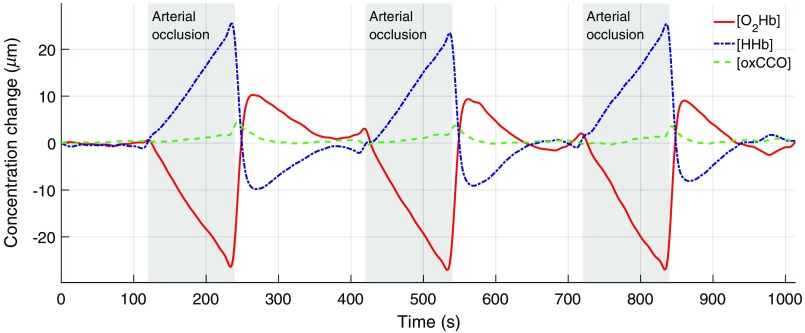
Arterial occlusion measurement with reconstructions of $\left[O_{2}\mathrm{Hb}\right]$ (red), [HHb] (blue), and [oxCCO] (green). Occlusions were performed three times (after 120, 420, and 720 s) and lasted 120 s each (gray areas).

Figure 5 shows the calculated concentration changes during an arm arterial occlusion. As expected, a small increase of $\left[O_{2}\mathrm{Hb}\right]$ and [HHb], originating from an incomplete occlusion (venous occlusion), is observed when the pressure cuff is inflated, which is followed by a simultaneous increase of $\left[O_{2}\mathrm{Hb}\right]$ and decrease of [HHb] (arterial occlusion). The concentration change of [oxCCO] is small in comparison with the hemoglobin changes; when blood flow is occluded, a small upward trend is visible with a change less than 1/10 of [HHb]. After pressure release from the cuff, the typical hyperemic change with a $\left[O_{2}\mathrm{Hb}\right]$ overshoot and a [HHb] undershoot can be measured. At the same time, [oxCCO] reveals a small peak (about 1/5 of [HHb] peak change).

#### 3.2.2 Task-evoked brain activity

**Fig. 6:**
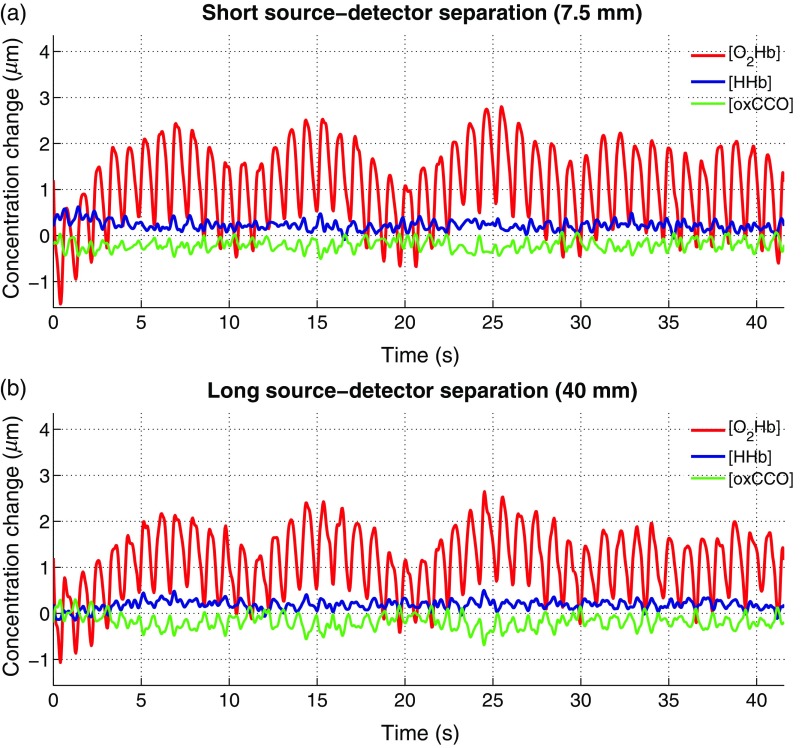
Concentration changes obtained from a resting brain state. (a) Chromophore changes for the SS channel over 40 s. (b) A simultaneous measurement with the LS channel is shown.

Figure 6 shows an example of resting state brain measurement with one SS and one LS channel. During rest, only extracerebral signal changes are expected, which is best seen in the $\left[O_{2}\mathrm{Hb}\right]$ signals. In $\left[O_{2}\mathrm{Hb}\right]$ of both channels, nearly identical signal contributions with the expected low-frequency oscillations (Mayer waves) and the heartbeat are measured.

**Fig. 7:**
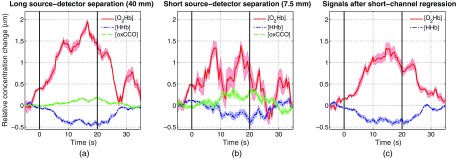
Block average of concentration changes during a motor execution task. Task duration was 20 s (black vertical lines) and reconstructions of $\left[O_{2}\mathrm{Hb}\right]$ (red), [HHb] (blue), and oxCCO (green) were performed (shaded area: standard error). (a) Measurement with LS channel (40 mm). (b) Measurement with SS channel (7.5 mm). (c) $\left[O_{2}\mathrm{Hb}\right]$ and [HHb] after the application of the short-channel regression method according to Saager and Berger.^19^

For the motor execution task, group averages for SS and LS channels were performed over the 10 repetitions (Fig. 7). During the task, a clear hemodynamic response with an increase of $\left[O_{2}\mathrm{Hb}\right]$ and a simultaneous smaller decrease in [HHb] was measured in the LS channel. [oxCCO] remained unchanged during the entire measurement. In the LS channel, task-related physiological changes can be observed, but no distinct hemodynamic response was obtained. After application of the short-channel regression to remove hemodynamic changes occurring in the extracerebral tissue layer (scalp blood flow) from $\left[O_{2}\mathrm{Hb}\right]$ and [HHb], a filtered signal with removed peaks at task onset (t=6  s) and during rest (t=36  s) was obtained.

## 4 Discussion

In this paper, the development and characterization of an fNIRS instrument was presented. It combines three innovative features, namely (i) the simultaneous measurement with SS and LS channels, (ii) the emission of NIR light at four wavelengths, and (iii) a modular optode arrangement to optimize the concentration signals that can be obtained from an fNIRS instrument. To the best of our knowledge, the proposed device is the first wearable fNIRS instrument implementing all these three key features. It further provides low-noise and fast data acquisition, thus maximizing the reliable determination of concentration changes at short and long SDS. Several improvements in comparison with our previous prototype^24^ were achieved, including minimization of temperature drift, implementation of $I^{2}C$ readout for fast data communication, design of safety circuits, advanced processing steps for the same sampling frequency, and miniaturization of the PCBs.

The instrument allows simultaneous measurements with a large optical dynamic range of 160 dB, outperforming other existing wearable fNIRS instruments (80 dB in Ref. 4, 55 dB in Ref. 6). Robust measurements (i.e., SNR>40  dB) can be obtained at OL up to $5\times 10^{6}$, corresponding to SDS of ∼50 to 55 mm in human head tissue.^30^ This finding was confirmed by a functional measurement on a human subject where a long SDS of 40 mm over the primary motor cortex was used, which is the same range or larger than SDS typically used in other fNIRS instruments (25 to 42 mm).^3^^,^^4^^,^^6^ According to simulations, increasing the SDS from 30 to 40 mm improves the sensitivity to detecting hemodynamic changes in the brain by ∼35%.^21^ In general, the obtained SNR values for longer SDS (47 dB at $10^{6}$ OL, 31 dB at $10^{7}$ OL) are comparable^24^^,^^26^^,^^30^ or slightly larger^4^^,^^32^ than those reported in the literature and are in the same range for shorter SDS with OL below $10^{4}$ (SNR of 64 dB). In comparison with our previous prototype,^24^ identical SNR values were obtained, except for short SDS (74 dB instead 64 dB) where the maximal photocurrent was decreased from 6 to 1 mA to reduce heating effects in the SiPM. The SNR at longer SDS could be further improved by increasing the LED emission power (e.g., up to 20 mW such as in Piper et al.^3^). Optical drift in the raw signal could be maintained at a small level with values below 0.1 ‰/s, which does not affect concentration calculation and is in the same magnitude as that reported in other work.^47^ The overall sampling frequency of 100 Hz is comparably high, reducing electronic noise due to oversampling and making measurements with multiple modules (e.g., up to 10 modules) possible.

The design of the presented prototype enables wearable applications thanks to the highly miniaturized hexagonal modules and a compact control unit supporting real-time wireless communication. The structure based on small size and low weight optodes that are connected to a control unit that can be placed in a backpack is similar to the approach presented by Piper et al.^3^ In comparison with other approaches,^4^^,^^6^^,^^48^ the modules placed on the head are distinctly smaller in our instrument, thereby decreasing the risk of uncomfortable optode placement and motion artefacts. Measurements with only one, but also with several modules—depending on the individual user needs and the application—are possible. When 10 modules are connected, a measurement with 100 channels and 6.25 Hz is achievable. By placing multiple modules next to each other, high-density optical channel arrangements are achievable, with a channel network similar to diffuse optical tomography (DOT) approaches.^49^^,^^10^ The DOT approach by Chitnis et al.^10^ provides the possibility to arrange four modules in such a way that a simultaneous 128-channel measurement with two wavelengths can be realized.

When additional wavelengths are included in the calculation of concentration changes, the influence of electronic noise can be reduced significantly, which was demonstrated on phantom measurements by a decrease in the variance of $\left[O_{2}\mathrm{Hb}\right]$ and [HHb] by 25% and 8%, respectively, when four instead of the typical two wavelengths were used. These results suggest that more accurate and robust chromophore calculations can be realized thanks to additional wavelengths, leading to more information content in the MBLL. Furthermore, when using four wavelengths, it is possible to calculate [oxCCO], which is known as a marker of mitochondrial oxygen consumption. This information might allow a more robust determination of brain activity since oxCCO should be independent of systemic signals,^22^ which bears high potential for increasing the accuracy of classifiers in BCI applications. Nevertheless, the use of oxCCO is controversially discussed, as no golden standard for *in vivo* validation measurements exists at the moment and its accurate calculation is influenced by several factors (e.g., LED emission spectra, the DPF values, temperature effects, reconstruction method, extinction spectra).^37^^,^^50^

A distinct and expected hemodynamic response during a finger-pinching task was measured in the LS channel over the primary motor cortex. The hemodynamic response showed the same behavior as reported in Refs. 46 and 51 (i.e., an increase in $\left[O_{2}\mathrm{Hb}\right]$ and a decrease in [HHb]). In the SS channel, no distinct hemodynamic response was observed (i.e., the decrease in [HHb] was absent), which goes along with expectations since we assume that little to no signal contributions from cerebral regions were measured. However, perfusion changes in the extracerebral tissue in response to the task^17^^,^^47^ were observed (i.e., a strong increase in $\left[O_{2}\mathrm{Hb}\right]$). The double-peak in [O2Hb] in the SS channel is also an indication for scalp blood flow changes in response to a mental task (own observation based on various experiments and measurement devices). By performing a short-channel regression according to Saager and Berger,^19^ a cleaner hemodynamic response was obtained, highlighting the feasibility and benefit of including a short SDS for filtering and demasking the LS channel from physiological noise. While other approaches for removing physiological noise exist, e.g., through software filtering applied to the LS channel only to reduce confounding effects (see Ref. 12 for a review), these algorithms are sophisticated, computationally demanding, and cannot make up for the additional information obtained when measuring systemic signals directly. SS channels deliver information that is important for a correct interpretation of fNIRS measurements. Zimmermann et al.^52^ proposed a different approach in acquiring biosignals in parallel (i.e., heart rate, breathing rate, blood pressure, and skin conductance). However, this comes at the disadvantage of requiring additional experimental setup that is complicated to use, time-consuming to set up, and, therefore, limited to laboratory use.

To further improve the proposed instrument, the replacement of the selected LED types should be considered to measure with more suitable optical properties (smaller FHWM, more accurate peak wavelengths^32^) and to minimize the distances between the four LEDs (e.g., using multiwavelength LEDs), thereby better satisfying the assumptions of the MBLL (i.e., point source, single emission wavelength). A mechanical casing that contains optical fibers for simpler guidance of the light through the hair (which is a common issue in all fNIRS instruments) should be developed. Careful selection of optical fibers should allow improvement of the robustness of the instrument for use in different experimental paradigms. In combination with a head fixation, for example, similar to available electroencephalography-setups such as the Emotiv EPOC (Emotiv, California), wearability and simple use of the device should be guaranteed. By providing a robust and unobtrusive module fixation, we hope to make the step out of the laboratory environment toward everyday environments with the proposed fNIRS instrument. In this work, no compensation for the nonlinear SiPM behavior at short SDS was performed. By acquiring calibration measurements at various short SDS, the nonlinearity could be compensated for, with potential to further improve estimations of concentration changes. In future applications, it is desirable to deploy and test a configuration with a larger number of modules to obtain simultaneous information from multiple brain areas and to generate two-dimensional images based on the measured signals.

## 5 Conclusion

An fNIRS instrument with three important features (i.e., short and long SDS, four-wavelength light sources, modular placement and configuration) was proposed and implemented. High modularity is achieved through miniaturized hardware design of optode modules that contain sources and detector and that can be individually connected to a central unit. The inclusion of SS and LS channels help to detect and compensate for physiological signals, a crucial feature for many fNIRS applications, in particular BCIs. By including four wavelengths, more robust estimates of concentration changes could be achieved, while allowing further investigation of the use of [oxCCO] as an additional marker for brain activity.

High-quality test bench measurements that outperform existing fNIRS instruments in many aspects were obtained, and *in vivo* tests confirmed the sensible chromophore calculation of $O_{2}\mathrm{Hb}$, HHb, and oxCCO. The proposed prototype could pave the way for robust fNIRS measurements in real-life applications.
